# A case report of atypical sleep in an ischemic stroke patient with psychiatric symptoms caused by olanzapine

**DOI:** 10.3389/fneur.2023.1266204

**Published:** 2023-12-21

**Authors:** Huixin Zhao, Shuang Li, Yanmei Wang

**Affiliations:** ^1^Department of Pharmacy, North China University of Science and Technology Affiliated Hospital, Tangshan, China; ^2^School of Public Health, North China University of Science and Technology, Tangshan, China; ^3^Medical Security Center, The PLA 982 Hospital, Tangshan, China

**Keywords:** cerebrovascular disease, olanzapine, anomalous sleep, adverse reactions, RBD (rapid-eye-movement sleep behavior disorder)

## Abstract

Antipsychotics, tricyclic and 5-hydroxytryptamine reuptake inhibitors (SSRI) and 5-hydroxytryptamine and norepinephrine reuptake inhibitor (SNRI) antidepressants, and monoamine oxidase inhibitors can produce dream-rendering behaviors and/or dystonic deregulation during REM sleep. Acute episodes are also seen with withdrawal from alcohol or sedative-hypnotics, and the use of tricyclic and SSRI antidepressants. In this article, we present a case of olanzapine treatment of a patient with cerebrovascular disease with psychobehavioural symptoms. The patient was an elderly patient who developed psychobehavioural symptoms after a sudden cerebral infarction. Initially, his symptoms improved when he took olanzapine (5 mg orally once/night). However, the patient subsequently developed symptoms of hypersomnia when he continued to take olanzapine, and the symptoms of hypersomnia gradually worsened as the dose of olanzapine was gradually increased. Benzodiazepines are often used to treat anomalous sleep, and clonazepam is one of the commonly prescribed drugs. In this case, the patient’s abnormal sleep behavior was alleviated after treatment with clonazepam. As an atypical antipsychotic drug, olanzapine has been reported to cause abnormal sleep behavior during clinical use, and only one case has been reported in China. Clinicians should be aware that heteromorphic sleep can occur in patients treated with olanzapine.

## Introduction

Cerebrovascular disease (CVD) is a common condition that is highly prevalent and often associated with psycho-behavioral symptoms. In the treatment of these symptoms, pharmacotherapy is the main treatment modality. The efficacy of olanzapine, a thiophene phenylpropanediazine (1), in patients with cerebrovascular disease with psychiatric-behavioral symptoms has been widely recognized by Chinese clinicians (1–3). However, there are fewer studies on the possible adverse effects of olanzapine and its effects on psychiatric symptoms.

Anomalous sleep is a combination of sleep disorders including arousal disorders, sleep transition disorders, anomalous sleep associated with REM sleep, and other anomalous sleep. Of these, Rapid Eye-Movement Sleep Behavior Disorder (RBD) is a common type of Anomalous sleep and is particularly common in older adults. The main feature of RBD is the presence of complex dreams and violent behavior during deep sleep. Although violent behavior in RBD usually lasts only a few seconds to a few minutes, it often leads to incidents of self-injury and injury. Some patients do not wake up as a result of these behaviors, although some recall the associated dreams upon awakening (4, 5).

Clinical studies have shown that olanzapine, as an atypical antipsychotic drug, can increase slow-wave sleep by blocking the 5-hydroxytryptamine 2C receptor and induce nocturnal arousal, thus leading to anomalous sleep (6, 7). However, there are fewer case reports of olanzapine-induced Anomalous sleep (7), probably because Anomalous sleep usually occurs at night and is not easily recognized, although its effects and dangers cannot be ignored. Based on this background, we report a case of a patient with cerebrovascular disease combined with psycho-behavioral symptoms, who developed Anomalous sleep after taking olanzapine, in order to draw clinicians’ attention to the side effects of this drug.

## Case presentation

The patient, an 82-year-old male, was admitted on April 4, 2023, presenting with sudden weakness in both lower limbs and a 2-hour episode of speechlessness. His medical history includes 44 years of hypertension, which is currently managed with losartan for effective blood pressure control. Additionally, he had cerebral infarction 34 years ago, with no significant residual symptoms. There is no record of viral hepatitis, tuberculosis, or other infectious diseases. The patient has no history of blood transfusion, drug allergies, or food allergies. Upon admission, various anicllary examinations were conducted, including complete blood count, electrolytes, liver and kidney function tests, cardiac enzymes, thyroid function test, coagulation function test, and viral marker tests. The results of these tests revealed no abnormalities. The cranial CT scan revealed infarction lesions in both hemispheres of the brain, hemodynamic changes in the carotid arteries and vertebral artery, and partial weakness on one side of the body, indicating involvement of both cerebral hemispheres. Considering the patient’s sudden onset, partial symptom relief, and occurrence of ischemic stroke within 48 h, the patient received treatment to promote collateral circulation, clear free radicals, and stabilize plaques and regulate lipids. This treatment was based on the “Expert Consensus on the Clinical Application of Ticagrelor in Atherosclerotic Cerebrovascular Diseases (8).” The patient’s condition was monitored based on the results of further examinations.

As of April 5th, the patient’s condition remained stable on the stroke treatment. The patient exhibited mental and behavioral symptoms during the night, including disrupted sleep–wake cycle, fear, and anxiety. Physical examination revealed clear consciousness, dysarthria, normal eye movements, reactive pupils, symmetrical nasolabial folds, no drooping of the corners of the mouth, midline tongue position, no deviation when opening the mouth, upper limb muscle strength at level 5, lower limb muscle strength at level 4+, increased muscle tension in the lower limbs, bilateral tendon reflex (++), negative meningeal signs, symmetrical facial and limb sensation, and no abnormalities in coordination tests. Follow-up cranial CT scan indicated bilateral multiple lacunar infarctions in the brain, with some lesions already softened, brain protein loosening, and brain atrophy. A consultation with a psychiatrist was advised, and olanzapine tablets (Olfen, 10 mg/tablet, Qilu Pharmaceutical Co., Ltd., 5 mg orally, once at night) were added. The patient’s psychiatric symptoms improved. On April 6th, the patient experienced restlessness, involuntary scratching of the wall with both hands, and continuous extension and flexion of the lower limbs while asleep. The patient had tightly closed eyes, continuous mumbling, and was unresponsive to others. The dosage of olanzapine tablets was temporarily increased to 10 mg, and the patient’s condition was closely monitored. On the night of April 7th, the patient continued to take olanzapine tablets (10 mg orally, once at night). During sleep, the patient exhibited delirium, agitation, punching and kicking, incoherent speech, and physical aggression. The patient damaged the bed rail and ceiling. Video polysomnography recorded showed these behaviors occurred during REM sleep. Diazepam injection was administered intravenously to induce sleep, and the patient had no recollection of the events the next day. On the morning of April 8th, a clinical pharmacist was consulted due to the patient’s abnormal nocturnal behavior. The clinical pharmacist determined that the patient’s symptoms during REM sleep, including delirium, agitation, punching and kicking, incoherent speech, and physical aggression, were consistent with rapid eye movement sleep behavior disorder (9). It was concluded that these symptoms were a drug adverse reaction to olanzapine. Olanzapine was discontinued, and the recommended medication treatment plan included clonazepam tablets (2 mg/tablet, Jiangsu Enhua Pharmaceutical Co., Ltd.) 0.5 mg orally before bedtime. If necessary, melatonin could be added. That night, the patient took clonazepam tablets and had symptomatic relief and went to sleep. From April 8th onwards, the treatment plan for abnormal sleep remained unchanged, and the patient did not exhibit the aforementioned behaviors. The psychotic symptoms also improved.

## Discussion

Based on the assessment of adverse reactions, it is important to note that olanzapine is commonly used as an atypical antipsychotic for the treatment of schizophrenia. In this case, the patient experienced abnormal sleep disturbances after taking olanzapine. The correlation between the adverse reaction and olanzapine is supported by four out of the five evaluation criteria outlined in the “Guidelines for the Use of Adverse Drug Reaction Terminology (10)”by the National Adverse Drug Reaction Monitoring Center. Firstly, the timing of the reaction aligns with the use of the drug, as the patient developed abnormal sleep symptoms after taking olanzapine. Secondly, the reaction is consistent with known adverse reactions of abnormal sleep, which have been associated with other drugs such as tricyclic antidepressants, SSRI, SNRI, resulting in REM sleep behavior disorder and REM sleep without atonia (RWA) (6). Thirdly, the patient’s abnormal sleep symptoms gradually improved and disappeared after discontinuing olanzapine, indicating that the reaction subsided with the cessation of the drug. Lastly, the response cannot be explained solely on the basis of comorbid medications or the patient’s existing medical condition. For the diagnosis of stroke-related RBD, the diagnostic criteria for both stroke and RBD must be met. However, according to the ICSD-3 diagnostic criteria (11), the patient did not fully meet this diagnostic requirement. There was no evidence of side effects from the patient’s previous medications that could have triggered anomalous sleep disturbances. In addition, the patient did not have a past history of heteromorphic sleep, such as sleepwalking or night terrors. Of note, the patient developed ectomorphic sleep within 24 h of starting olanzapine, which was closely related to the timing of drug intake. The abnormal behavior exhibited by the patient during sleep was associated with olanzapine and the patient had no memory of this upon awakening (12), which is consistent with the known symptoms of olanzapine. Following the administration of clonazepam, the patient’s abnormal sleep behavior resolved, which further supports the association with olanzapine. Therefore, it can be reasonably determined that there is a high likelihood of an association between the patient’s abnormal sleep adverse reactions and olanzapine.

### Mechanisms by which olanzapine causes anomalous sleep

Olanzapine is a thiophene phenylpropanediazine that belongs to the second generation of atypical antipsychotics. It has a high degree of multireceptor blockade, including blockade of 5-hydroxytryptamine receptors, dopamine D2 receptors, and norepinephrine (1). Olanzapine tends to strongly block 5-HT2A receptors compared to the blocking effect on dopamine D2 receptors (1, 13). Studies have shown that olanzapine, as an atypical antipsychotic drug, can increase slow-wave sleep by blocking the 5-hydroxytryptamine 2C receptor and induce nocturnal arousal, thus leading to anomalous sleep (7).

Anomalous sleep, also known as RBD, is a common type of sleep disorder in the elderly, which is mainly characterized by complex dreams and violent behavior during deep sleep. RBD is often characterized by violent behavior that lasts for a few seconds to a few minutes, and often results in self-injury and injuries, and patients do not wake up from the violent behavior, but some of them can recall the related dreams after waking up (4, 5). A number of drug-induced RBDs have been reported to date, such as antipsychotics, tricyclic antidepressants, SSRIs, and SNRIs, which have been associated with the development of RBD symptoms as well as loss of muscle relaxation in the REM phase without dream rendition. Other drugs, such as monoamine oxidase inhibitors, beta-blockers, and cholinesterase inhibitors have also been reported to induce RBD episodes and should be brought to the attention of clinical providers.

### Symptoms and treatment of anomalous sleep

A common treatment for RBD is the use of benzodiazepines, of which clonazepam is one of the commonly prescribed drugs. Clonazepam is a long-acting benzodiazepine that has a rapid onset of action and can significantly inhibit abnormal behaviors in patients with RBD, and has long been used as a first-line drug for RBD (14). The effectiveness of clonazepam in the treatment of RBD has been first reported in 1986 (15). Currently, the mechanism of action of clonazepam in the treatment of RBD is unclear.

Melatonin, a hormone secreted by the pineal gland with increased nocturnal secretion (16), has a similar therapeutic effect on RBD as clonazepam with fewer adverse effects (17). Therefore, melatonin is often used as an alternative drug in clonazepam-intolerant individuals. However, the mechanism of action of melatonin in the treatment of RBD is still unclear. It has been suggested that the therapeutic effects of melatonin may be mediated by a combination of influences, including direct effects on REM sleep relaxation disorder, modulation of γ-aminobutyric acidergic inhibition, stabilization of circadian rhythm variability and desynchronization, and improvement of sleep efficiency (18, 19).

Currently, most of the research on pharmacological treatment of RBD comes from observational studies and a few randomized controlled trial studies. In these studies, traditional medications represented by clonazepam and melatonin are supported by more evidence in terms of efficacy, while safe sleep environments remain the preferred non-pharmacological treatment. When using medications to treat RBD, older patients should be started on small doses.

In summary, Although the atypical antipsychotic drug olanzapine is widely used in clinical practice, its high incidence of adverse reactions has a direct impact on the safety of medication and clinical efficacy of patients (20). In China, reports of rare RBD adverse reactions are extremely limited, which means that clinical healthcare professionals need to be more vigilant and pay more attention to this problem. To ensure patient safety and improve clinical efficacy, we strongly recommend that more studies be conducted to gain a deeper understanding of olanzapine-associated adverse reactions and to provide proper preventive and therapeutic strategies. This will help to improve the rational use of olanzapine and reduce the risk of adverse effects in patients, thereby promoting recovery and quality of life.

## Data availability statement

The original contributions presented in the study are included in the article/supplementary material, further inquiries can be directed to the corresponding author.

## Ethics statement

Ethical review and approval was not required for the study on human participants in accordance with the local legislation and institutional requirements. Written informed consent from the patients/participants or patients/participants’ legal guardian/next of kin was not required to participate in this study in accordance with the national legislation and the institutional requirements. Written informed consent was obtained from the individual(s) for the publication of any potentially identifiable images or data included in this article.

## Author contributions

HZ: Formal analysis, Investigation, Writing – original draft, Writing – review & editing. SL: Supervision, Validation, Writing – review & editing. YW: Investigation, Validation, Writing – review & editing.
